# Association of self-esteem, personality, stress and gender with performance of a resuscitation team: A simulation-based study

**DOI:** 10.1371/journal.pone.0233155

**Published:** 2020-05-14

**Authors:** Lucas Tramèr, Christoph Becker, Cleo Schumacher, Katharina Beck, Franziska Tschan, Norbert K. Semmer, Seraina Hochstrasser, Stephan Marsch, Sabina Hunziker

**Affiliations:** 1 Medical Intensive Care Unit, University Hospital Basel, University of Basel, Basel, Switzerland; 2 Medical Communication and Psychosomatic Medicine, University Hospital Basel, University of Basel, Basel, Switzerland; 3 Department of Emergency Medicine, University Hospital Basel, University of Basel, Basel, Switzerland; 4 Department of Psychology, University of Neuchâtel, Neuchâtel, Switzerland; 5 Department of Psychology, University of Bern, Bern, Switzerland; NASA Johnson Space Center, UNITED STATES

## Abstract

**Background:**

Gender composition, stress and leadership of a resuscitation team influence CPR performance. Whether psychological variables such as self-esteem, motivation and personality traits are associated with resuscitation performance, stress levels and gender of rescuers during a cardiac arrest scenario remains uncertain.

**Methods:**

We included 108 medical students in this prospective, observational simulator study. We videotaped the resuscitation performance and assessed self-esteem, perceived stress-overload and personality traits using validated questionnaires. In addition, we analysed leadership utterances and ECG data of all participants during the simulation. The primary endpoint was cardiopulmonary resuscitation performance, defined as hands-on time within the first 180 sec. Secondary outcomes included first meaningful measure of resuscitation, leadership statements of group leaders and physiological stress parameters of rescuers.

**Results:**

Adjusted for group size and leadership designation, mean self-esteem of students was significantly associated with hands-on time (adjusted regression coefficient 7.94 (95%CI 2.61 to 13.27), p<0.01). The personality trait conscientiousness was positively associated with hands-on time (adjusted regression coefficient 38.4, [95%CI 7.41 to 69.38, p = 0.02]). However, after additional adjustment for self-esteem, this association was no longer significant. Further, agreeableness of team leaders was significantly associated with longer hands-on time (adjusted regression coefficient 20.87 [95%CI 3.81 to 37.94], p = 0.02). Openness to experience was negatively associated with heart rate reactivity (-5.92 (95%CI -10 to -1.85), p<0.01). Male students showed significantly higher (mean, [±SD]) self-esteem levels (24.6 [±3.8] vs. 22.0 [±4.4], p<0.01), expressed significantly more leadership statements (7.9 [±7.8] vs. 4.6 [±3.8], p<0.01) and initiated first resuscitation measures more often (n, [%]) compared to female students (16, [23] vs. 7, [12], p = 0.01).

**Conclusion:**

This simulator study found that self-esteem of resuscitation teams and agreeableness of team leaders of inexperienced students was associated with cardiopulmonary resuscitation performance. Whether enhancing these factors during resuscitation trainings serve for better performance remains to be studied.

## Introduction

Witnessing a cardiac arrest causes serious mental stress among rescuers [1]. Psychological stress may be defined as a physical as well as an emotional response to the feeling of strain and pressure caused by an external stimulus[2]. The effect of psychological stress on cardiopulmonary resuscitation (CPR) performance remains unknown with current studies reporting conflicting results [1, 3–5]. Already in 1985, Lazarus described that performance could be either enhanced or impaired by stress, depending on the individual’s coping mechanisms and perception of the task to perform[6]. Consequently, coping with mental stress might reflect the individual’s personality and its adaptation to perceived external pressure. Most likely, the relationship between mental stress and CPR performance is mediated by other variables. Previous studies found associations between personality traits and self-esteem with stress response.[7–10]. Especially the personality traits openness to experience, extraversion and conscientiousness are thought to be associated with active coping mechanisms leading to reduced stress levels of individuals[10–12]. People with high self-esteem, a factor that is associated with lower stress-levels through adaptive strategies, may be more willing to speak up in groups, show more proactive behaviour and may dare to criticize a group’s approach[13]. There is evidence that such leadership utterances have a positive impact on CPR performance [14–16].

Moreover, some studies have demonstrated important gender differences in psychological variables, with men having higher levels of self-esteem[17]. Also, gender research suggests differences in personality traits with higher levels of neuroticism in women [18–21]. In a medical setting, Amacher et al. found that male students made more secure leadership statements (e.g. “start CPR now!”, in comparison with insecure statements: “Should we give epinephrine?”) and showed better performance in a simulated CPR setting than their female colleagues. [22].

However, there is a lack of clinical studies evaluating the impact of psychological variables on resuscitation performance. The aim of our study was to investigate the association of personality traits, self-esteem and motivation with resuscitation performance, leadership statements and physiological stress markers of medical students during a simulated cardiac arrest.

**Fig 1:**
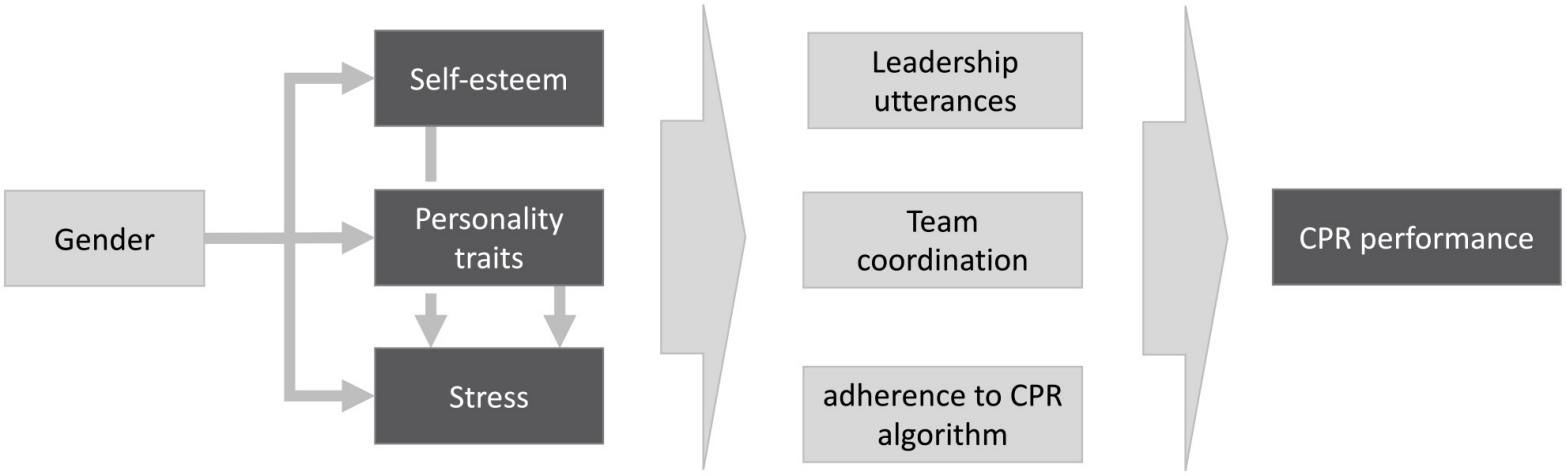
Hypothesized relationships between gender, psychological variables and CPR performance.

The main hypothesis of this study was that personality, self-esteem and stress are associated with CPR performance (Fig 1). We anticipated that extraversion, openness to experience as well as high self-esteem have a performance enhancing effect during a CPR scenario and trigger leadership utterances. These were divided into secure and insecure utterances as we were interested in finding if psychological variables, alike gender, have an impact on the security of leadership utterances. Further, we hypothesized that the psychological variables of designated team leaders would have an effect on team performance. In addition, we expected that psychological variables would be associated with subjective and electrocardiographic stress parameters. Finally, we expected gender differences with male students showing higher levels of self-esteem, openness to experience and lower levels of neuroticism compared to female students.

## Methods

### Study design and participants

This is a secondary analysis of a prospective, observational study performed between December 2011 and April 2012 at the Simulator Centre in Basel, Switzerland [23]. A total of 126 medical students (70 female, 56 male) of the University of Zurich participated in a simulated CPR scenario. The students were randomly allocated into groups of 3 or 6 students. Complete data regarding students’ self-esteem, perceived stress-overload and personality traits were available for 108 medical students in 23 groups and thus included in this analysis. In 11 out of the 23 groups, a team-leader was randomly designated prior to the simulation. The study was conducted for both research and educational purposes. Each team only participated once. The video recording of the resuscitation was shown to each team immediately after the simulation and a constructive debriefing was held. The study was approved by the local Ethics Committee of Northwest and Central Switzerland (EKNZ). All students gave written informed consent.

### Simulator

A high-fidelity mannequin with the possibility of remote control of vital signs was used (Human Patient Simulator, METI, Sarasota, FL, USA) to allow realistic interactions.

### Scenario and study protocol

Prior to the simulation, participants were asked to complete a self-esteem questionnaire (Rosenberg self-esteem scale [24], see below) and a personality questionnaire (NEO Big Five Inventory [25, 26], see below). All students were equipped with a 3-lead Holter ECG device which recorded during the whole simulation. During the simulation, the participants were asked to quantify their perceived levels of motivation and stress-overload.

Participants were allocated into teams of three or six rescuers. Each team was only tested once. In eleven groups, a randomly selected participant was assigned the role of group leader (“leadership designation”). The other rescuers were asked to follow instructions given by the group leader during the resuscitation scenario. Each team was asked to handle the same scenario, in which a patient faints because of a pulseless ventricular tachycardia. The cardiac arrest lasted for at least 3 minutes independent of the actions taken by the team. In every group, the simulated patient survived the cardiac arrest. One person, blinded to allocation, was trained and rated the video-recordings using the adapted Leader Behavior Description Questionnaire (LBDQ)[27–29]. The LBDQ is a frequently used and evaluated tool to assess leadership behaviour. CPR performance and leadership statements were coded second-by-second using video-recordings of the simulation.

### Resuscitation performance and leadership statements

According to the current CPR Guidelines, early start of CPR and uninterrupted chest compressions are crucial for patient survival[30]. Thus, we used these two variables to assess CPR performance using the video-recordings of the simulation. First, we assessed total hands-on time (uninterrupted CPR) during the first 3 minutes after the onset of the cardiac arrest. Second, we determined the first meaningful measure of resuscitation (FMM) for each group, defined as the time elapsed until CPR was started after the onset of cardiac arrest (either defibrillation, chest compression or ventilation). Smaller FMM values reflect faster action and thus better CPR performance.

As we hypothesized that psychological variables would be associated with leadership statements, we assessed leadership statements of all participants using an adapted form of the Leadership Behaviour Description Questionnaire (LBDQ) previously used by Cooper and Wakelam [14, 28] in the analysis of leadership in resuscitation. The following utterances and actions were coded: (1) task assignment, (2) decision about what should be done, (3) decision on how things should be done, (4) specific command, (5) meaningful measure without comment (e.g. starting CPR without commenting about it), (6) scheduling of work to be done, (7) making sure the leader-role is understood, (8) specific correction. In line with previous research, leadership statements were divided into secure (i.e., strong and direct statement “use the defibrillator!”) and insecure (i.e., formulated as a question “should we defibrillate?”) utterances [22].

### Electrocardiographic measurements

Employing a 3-lead Holter ECG device (Philips Zymed Holter Serie 1810 2.9.2), we assessed heart rate (HR) and heart rate variability (HRV) as markers of supraventricular sympathetic drive. During mental stress, HR increases and HRV decreases as a consequence of increased sympathetic drive [31, 32]. We calculated the SDNN (standard deviation of heart beats intervals) as a marker for total heart rate variability and RMSSD (mean square difference of heart beat intervals) as a marker of parasympathetic tone [33]. Additionally, we assessed dynamic ECG changes such as decreases of T-wave amplitude (TWA) or variations of the ST segment, typical for myocardial stress [31]. Measurements were started 10min before and ended 30min after the end of the scenario. HR, SDNN and RMSSD were averaged over the course of the scenario.

### Assessment of self-esteem: Rosenberg self-esteem scale (RSE)

The Rosenberg self-esteem scale (RSE) is a self-report instrument for the evaluation of self-esteem [24, 34]. The scale measures the participants’ perception of their own worth on a scale containing ten self-descriptive statements, of which five are positively worded and five are reverse worded. Each item is scored on a four point Likert scale ranging from “strongly agree” to “strongly disagree” [34]. Using a validated German translation of the RSE, we calculated a global self-esteem score based on the addition of the positively worded items and the reverse-scored negatively worded items, as recommended in previous literature [35, 36]. According to usual practice in previous studies, the RSE score was treated as a continuous variable for regression analysis [34, 37, 38]. For subgroup analysis, a median split was performed on the RSE score, creating a dichotomous self-esteem variable (high and low self-esteem), as used by Hughes et al. [39, 40].

### Assessment of personality traits: NEO Five Factor Inventory (Big Five)

To assess the personality structure of the participants we used a validated German version of the NEO Five-Factor Inventory tool [41]. The “Big Five” uses 30 items on a Likert scale from 1 to 6 to assess neuroticism, extraversion, openness to experience, agreeableness, and conscientiousness [15, 42]. The scores were kept on a continuous scale according to usual practice [7, 43, 44].

### Assessment of perceived stress-overload and motivation

During the resuscitation scenario, we assessed levels of feeling stressed, motivated, and overwhelmed on a Likert scale from 1 to 10. As feeling stressed and overwhelmed correlated significantly, we merged both variables into a “stress/overload” index by taking the average of both values, in line with previous studies[3, 4].

### Outcomes and their measurement

The primary outcome of our study was hands-on time, defined as uninterrupted chest compressions and defibrillation within the first 180 seconds after onset of cardiac arrest.

As a secondary outcome we assessed the first meaningful measure of resuscitation (FMM). Further secondary endpoints were leadership statements made by group leaders and ECG stress parameters such as heart rate, heart rate variability and dynamic ST-segment and T-wave alterations.

Finally, in a subgroup analysis we stratified the analysis based on gender, self-esteem and leadership designation.

### Data analysis

Individual differences across binary subgroups (e.g. self-esteem vs. gender) were compared using paired t-tests. To assess the association of self-esteem, perceived stress-overload and personality traits with CPR performance we performed linear regression analyses. For these analyses, the mean values of psychological variables amongst the members of each team were analysed in relation to team performance with hands-on time and FMM as linear group-level outcomes. As we expected designated team leaders to have an impact on team performance, the analysis of group leaders was performed using individual psychological variables in relation to group performance (e.g. self-esteem of leader vs. hands-on-time). Linear regression analyses were also performed to assess the association of ECG stress parameters and leadership statements with psychological variables.

All analyses were performed using STATA 15 (Stata Corp, College Station, TX). P values < 0.05 were considered to indicate statistical significance.

## Results

We included 108 medical students in 23 groups in this analysis. There were 58 female and 50 male students in their 4^th^year of medical school.

### Association of psychological variables with CPR performance

Regarding the primary endpoint on a group level, the mean (±SD) hands-on time in the first 180sec after onset of cardiac arrest was 94.6 sec (±25.5). CPR was started after a mean (±SD) of 59.3 sec (±17.8) and all groups successfully performed CPR. The simulation lasted for 283 seconds on average.

First, we investigated the association of different psychological variables within each team (i.e. self-esteem, personality, perceived stress-overload and motivation) with our primary endpoint, hands-on time (Table 1).

**Table 1:** Association of psychological variables of teams with CPR performance.

	Overall	Hands-on time during first 180s				First meaningful measure (FMM)			
	mean (SD)	Multivariate regression coefficient (95%CI)*	p-value	Multivariate regression coefficient (95%CI)**	p-value	Multivariate regression coefficient (95%CI)*	p-value	Multivariate regression coefficient (95%CI)**	p-value
**Self-esteem**									
Rosenberg self-esteem score	23.4 (1.9)	7.94 (2.61, 13.27)	**<0.01**			-3.45 (-7.7, 0.81)	0.11		
**Personality (Big Five)**									
Neuroticism	2.8 (0.4)	-20.19 (-52.86, 12.49)	0.21	12.46 (-25.07, 49.99)	0.49	7.19 (-16.31, 30.68)	0.53	-8.19 (-38.29, 21.91)	0.58
Extroversion	4.2 (0.3)	13.52 (-37.77, 64.82)	0.59	4.17 (-39.66, 48)	0.84	-0.59 (-36.62, 35.44)	0.97	3.59 (-31.4, 38.58)	0.83
Openness to experience	4.3 (0.3)	-5.66 (-50.07, 38.75)	0.79	-4.32 (-41.6, 32.95)	0.81	7.54 (-23.25, 38.34)	0.61	6.96 (-22.65, 36.58)	0.63
Agreeableness	4.6 (0.4)	-2.16 (-35.94, 31.63)	0.9	-7.69 (-36.04, 20.66)	0.58	9.19 (-13.95, 32.33)	0.42	11.74 (-10.36, 33.83)	0.28
Conscientiousness	4.4 (0.4)	38.4 (7.41, 69.38)	**0.02**	24.3 (-6.81, 55.41)	0.12	-18 (-41.59, 5.6)	0.13	-12.17 (-38.12, 13.78)	0.34
**Emotions during resuscitation**									
Stress-overload index	6.9 (1.2)	8.59 (-2.57, 19.75)	0.12	3.31 (-7.48, 14.1)	0.53	-3.19 (-11.34, 4.96)	0.42	-0.8 (-9.5, 7.9)	0.85
Perceived Stress	6.5 (1.2)	8.03 (-1.49, 17.55)	0.09	5.08 (-3.49, 13.66)	0.23	-3.84 (-10.75, 3.08)	0.26	-2.58 (-9.6, 4.44)	0.45
Perceived Motivation	8.4 (0.8)	1.96 (-13.62, 17.54)	0.8	-1.64 (-14.95, 11.67)	0.80	-2.65 (-13.46, 8.15)	0.61	-1.15 (-11.79, 9.48)	0.82

*adjusted for group size and group leadership designation

**adjusted for group size, group leadership designation and Rosenberg self-esteem score

CPR: cardiopulmonary resuscitation

In a first multivariate model, adjusted for group size and leadership designation, average group self-esteem among students was significantly associated with hands-on time (adjusted regression coefficient 7.94 (95%CI 2.61 to 13.27), p<0.01). The personality trait conscientiousness was positively associated with hands-on time (adjusted regression coefficient 38.4, 95% CI 7.41 to 69.38, p = 0.02) while stress-overload showed no association with hands-on time (adjusted regression coefficient 8.59, 95% CI -2.57 to 19.75, p = 0.12). No significant associations between psychological variables and hands-on time persisted after additional adjustment for self-esteem. The personality traits neuroticism, agreeableness, extroversion and openness to experience as well as motivation did not show any significant results.

Second, we investigated the association of psychological variables on the time elapsed until CPR was started after onset of cardiac arrest, defined as the first meaningful measure of resuscitation (FMM). Self-esteem showed a performance-enhancing (i.e., negative) association with FMM, however not reaching statistical significance (adjusted regression coefficient -3.45, 95% CI -3.45, 95% CI -7.7 to 0.81, p = 0.11). The other psychological variables showed no significant association with FMM.

### Association of psychological variables of group leaders with CPR performance and leadership statements

In 11 groups, a participant was randomly designated to be the group leader (7 men, 4 women). We investigated associations of psychological variables of group leaders with team performance and leadership statements (Table 2). Teams with an agreeable leader showed better performance with more hands-on time, which persisted after adjustment for group size, gender of leader and self-esteem of leader (adjusted regression coefficient 20.87, 95%CI 3.81 to 37.94, p = 0.02).

**Table 2:** Association of psychological variables of group leaders with CPR performance.

	Overall	Hands-on time during first 180s				First meaningful measure (FMM)				Secure leadership statements				Insecure leadership statements			
	mean (SD)	Multivariate regression coefficient (95%CI)*	p-value	Multivariate regression coefficient (95%CI)**	p-value	Multivariate regression coefficient (95%CI)*	p-value	Multivariate regression coefficient (95%CI)**	p-value	Multivariate regression coefficient (95%CI)*	p-value	Multivariate regression coefficient (95%CI)**	p-value	Multivariate regression coefficient (95%CI)*	p-value	Multivariate regression coefficient (95%CI)**	p-value
**Self-esteem**																	
Rosenberg self-esteem score	23.5 (3.9)	0.27 (-5.24, 5.79)	0.91			0.21 (-4.59, 5)	0.92			0.16 (-2.01, 2.33)	0.87			-0.3 (-0.67, 0.08)	0.11		
**Personality (Big Five)**																	
Neuroticism	2.8 (0.8)	-11.56 (-33.63, 10.52)	0.26	-25.82 (-59.5, 7.85)	0.11	7.81 (-12.19, 27.82)	0.39	20.67 (-9.88, 51.23)	0.15	-1.82 (-11.29, 7.64)	0.66	-3.14 (-19.53, 13.25)	0.66	1.74 (0.43, 3.05)	**0.02**	1.79 (-0.48, 4.07)	0.10
Extroversion	4.3 (0.8)	0.68 (-20.8, 22.16)	0.94	0.41 (-24.59, 25.42)	0.97	13.63 (-0.52, 27.79)	0.06	14.57 (-1.59, 30.72)	0.07	-5.83 (-12.5, 0.85)	0.08	-6.52 (-13.9, 0.86)	0.07	0.84 (-0.78, 2.47)	0.26	1.27 (0.12, 2.41)	**0.04**
Openness to experience	4.3 (0.5)	-24.33 (-62.86, 14.19)	0.18	-27.83 (-71.88, 16.22)	0.17	9.53 (-27.97, 47.02)	0.57	10.01 (-34.16, 54.18)	0.60	-7.13 (-23.35, 9.1)	0.33	-8.37 (-27.08, 10.35)	0.32	-0.2 (-3.88, 3.48)	0.90	0.62 (-2.89, 4.12)	0.68
Agreeableness	4.5 (0.8)	20.7 (5.5, 35.91)	**0.02**	20.87 (3.81, 37.94)	**0.02**	-5.01 (-25.34, 15.32)	0.58	-5.2 (-28.04, 17.65)	0.60	-3.29 (-12.25, 5.67)	0.41	-3.43 (-13.46, 6.61)	0.44	-0.69 (-2.59, 1.21)	0.42	-0.54 (-2.3, 1.22)	0.48
Conscientiousness	4.3 (1.0)	12.55 (-9.13, 34.22)	0.21	13.42 (-11.04, 37.88)	0.23	3.18 (-17.84, 24.2)	0.73	3.53 (-20.46, 27.53)	0.73	-3.67 (-12.7, 5.37)	0.37	-3.69 (-14.03, 6.65)	0.42	-0.62 (-2.58, 1.34)	0.48	-0.94 (-2.6, 0.72)	0.21
**Emotions during resuscitation**																	
Stress-overload index	7.1 (1.7)	6.19 (-2.57, 14.96)	0.14	7.83 (-2.29, 17.96)	0.11	1.13 (-7.82, 10.08)	0.77	1.58 (-9.43, 12.59)	0.74	-1.16 (-5.11, 2.79)	0.51	-1.26 (-6.13, 3.61)	0.55	0.21 (-0.64, 1.05)	0.58	-0.03 (-0.91, 0.84)	0.93
Perceived Stress	6.7 (2.0)	5.41 (-2.79, 13.62)	0.16	7.53 (-2.17, 17.24)	0.11	0.14 (-8.14, 8.42)	0.97	0.42 (-10.25, 11.09)	0.93	-0.93 (-4.59, 2.73)	0.57	-1.06 (-5.78, 3.66)	0.60	0.18 (-0.6, 0.96)	0.61	-0.1 (-0.94, 0.73)	0.77
Perceived Motivation	9.1 (1.0)	-2.28 (-24.66, 20.11)	0.82	-3.66 (-32.05, 24.74)	0.76	-1.64 (-21.12, 17.84)	0.85	-2.66 (-27.41, 22.09)	0.80	4.2 (-3.82, 12.22)	0.26	5.07 (-5, 15.13)	0.26	-0.53 (-2.34, 1.29)	0.51	0.07 (-1.89, 2.02)	0.94

*adjusted for group size and gender of leader

**adjusted for group size, gender of leader and self-esteem of leader

CPR, cardiopulmonary resuscitation

Extroverted leaders showed worse performance with longer time to start CPR without reaching statistical significance (adjusted regression coefficient 14.57, 95%CI -1.59 to 30.72, p = 0.07) and had significantly more insecure leadership statements (adjusted regression coefficient 1.27, 95% CI 0.12 to 2.41, p = 0.04). The remaining psychological variables of group leaders including self-esteem showed no association with hands-on time, FMM or leadership statements.

#### Associations of psychological variables with ECG recordings

On an individual level, the univariate associations of psychological variables with ECG recordings are summarised in Table 3. Thirty-six of our 108 students (33.3%) showed dynamic ECG alterations (T-wave or ST-segment). These alterations showed a positive association with the personality trait neuroticism (regression coefficient 0.12, 95% CI 0 to 0.24, p = 0.047).

**Table 3:** Association of psychological variables of individuals with ECG stress parameters (univariate regression).

	Heart rate						Heart rate variability				Dynamic ECG alterations	
	Mean HR [/min] regression coefficient (95%CI)	p-value	Maximal HR [/min] regression coefficient (95%CI)	p-value	HR reactivity [/min] regression coefficient (95%CI)	p-value	SDNN [ms] regression coefficient (95%CI)	p-value	RMSSD [ms] regression coefficient (95%CI)	p-value	alteration yes/no regression coefficient (95%CI)	p-value
**Self-esteem**												
Rosenberg self-esteem score	-0.31 (-1.13, 0.51)	0.46	-0.33 (-1.22, 0.56)	0.47	0.03 (-0.54, 0.61)	0.91	1.36 (-0.55, 3.27)	0.16	0.32 (-1.86, 2.5)	0.77	-0.01 (-0.03, 0.01)	0.33
**Personality (Big Five)**												
Neuroticism	3.27 (-1.52, 8.06)	0.18	2.86 (-2.36, 8.09)	0.28	-0.72 (-4.09, 2.65)	0.67	-7.75 (-18.98, 3.48)	0.17	-0.82 (-13.6, 11.96)	0.90	0.12 (0, 0.24)	**0.047**
Extroversion	-4.65 (-10.1, 0.79)	0.09	-3.63 (-9.6, 2.34)	0.23	-1.2 (-5.05, 2.65)	0.54	-3.03 (-15.98, 9.93)	0.64	-8.67 (-23.18, 5.85)	0.24	0 (-0.14, 0.14)	0.99
Openness to experience	-5.85 (-11.82, 0.12)	0.06	-8.36 (-14.76, -1.96)	**0.01**	-5.92 (-10, -1.85)	**<0.01**	-4.59 (-18.83, 9.66)	0.52	-5.61 (-21.66, 10.44)	0.49	-0.06 (-0.21, 0.09)	0.47
Agreeableness	5.88 (0.81, 10.94)	**0.02**	7.15 (1.68, 12.62)	**0.01**	1.87 (-1.74, 5.48)	0.31	-6.47 (-18.6, 5.65)	0.29	-9.45 (-23.06, 4.17)	0.17	-0.01 (-0.15, 0.13)	0.88
Conscientiousness	0.98 (-3.87, 5.84)	0.69	1.31 (-3.97, 6.59)	0.62	0.35 (-3.05, 3.74)	0.84	-2.37 (-13.76, 9.03)	0.68	-3.02 (-15.86, 9.82)	0.64	0.08 (-0.04, 0.2)	0.20
**Emotions during resuscitation**												
Stress-overload index	-0.76 (-2.62, 1.1)	0.42	-1.11 (-3.13, 0.9)	0.28	-0.9 (-2.19, 0.39)	0.17	-2.11 (-6.46, 2.25)	0.34	-2.85 (-7.75, 2.04)	0.25	-0.02 (-0.07, 0.03)	0.36
Perceived Stress	-0.1 (-1.88, 1.68)	0.91	-0.47 (-2.41, 1.46)	0.63	-0.69 (-1.93, 0.54)	0.27	-2.61 (-6.76, 1.53)	0.21	-3.0 (-7.68, 1.67)	0.21	-0.02 (-0.07, 0.03)	0.41
Perceived Motivation	-0.9 (-3.35, 1.55)	0.47	-1.54 (-4.19, 1.12)	0.25	-0.34 (-2.06, 1.37)	0.69	-1.58 (-7.34, 4.17)	0.59	-7.12 (-13.45, -0.79)	**0.03**	-0.04 (-0.1, 0.03)	0.24

SDNN: standard deviation of all normal to normal intervals; RMSSD: root-mean-square of successive differences; ms: milliseconds; Tachycardia: HR >100bpm; ECG: electrocardiogram

Heart rate parameters were significantly associated with the following personality traits: openness to experience was negatively associated with maximal heart rate (regression coefficient -8.36, 95%CI -14.76 to -1.96, p = 0.01) and heart rate reactivity (regression coefficient -5.92, 95%CI -10.0 to -1.85, p<0.01). Agreeableness was positively associated with maximal heart rate (regression coefficient 7.15, 95% CI 1.68 to 12.62, p = 0.01) and mean heart rate (regression coefficient 5.88, 95% CI 0.81 to 10.94, p = 0.02). Finally, motivation was negatively associated with short-term HRV (RMSSD) (regression coefficient -7.12, 95% CI -13.45 to -0.79, p = 0.03). No significant associations were observed between ECG stress parameters, perceived stress, stress-overload index or self-esteem levels.

*Association of psychological variables and leadership with gender and self-esteem*. Male students showed significantly higher levels of self-esteem than females (mean, [±SD]) (24.6 [±3.8] vs. 22.0 [±4.4], p<0.01) (Table 4). This association remained significant after adjustment for leader-designation.

**Table 4:** Subgroup analysis of psychological variables of individuals and leadership.

		Gender			Self-esteem			Leadership designation		
	Overall	Male	Female	p-value	High self-esteem	Low self-esteem	p-value	Non-leader	Leader	p-value
	n = 108	n = 50	n = 58		n = 50	n = 58		n = 97	n = 11	
**Self-esteem, mean (SD)**										
Rosenberg self-esteem score	23.2 (4.3)	24.6 (3.8)	22.0 (4.4)	**<0.01**	26.7 (1.4)	20.1 (3.6)	**<0.01**	23.1 (4.4)	23.5 (3.9)	0.76
**Personality (Big Five), mean (SD)**										
Neuroticism	2.9 (0.8)	2.6 (0.6)	3.1 (0.7)	**<0.01**	2.5 (0.6)	3.2 (0.7)	**<0.01**	2.9 (0.7)	2.8 (0.8)	0.95
Extroversion	4.2 (0.6)	4.1 (0.7)	4.3 (0.6)	0.09	4.3 (0.7)	4.1 (0.6)	0.14	4.2 (0.6)	4.3 (0.8)	0.64
Openness to experience	4.3 (0.6)	4.3 (0.7)	4.2 (0.5)	0.45	4.4 (0.6)	4.1 (0.6)	**0.02**	4.3 (0.6)	4.3 (0.5)	0.87
Agreeableness	4.6 (0.7)	4.6 (0.6)	4.6 (0.7)	0.99	4.7 (0.6)	4.5 (0.7)	0.05	4.6 (0.7)	4.5 (0.8)	0.55
Conscientiousness	4.4 (0.7)	4.3 (0.8)	4.5 (0.7)	0.15	4.5 (0.8)	4.4 (0.7)	0.45	4.4 (0.7)	4.3 (1.0)	0.48
**Emotions during resuscitation, mean (SD)**										
Stress-overload index	6.7 (1.8)	6.8 (1.9)	6.7 (1.8)	0.87	6.8 (1.8)	6.6 (1.9)	0.49	6.7 (1.9)	7.1 (1.7)	0.48
Perceived Stress	6.4 (2.0)	6.5 (1.9)	6.3 (2.0)	0.46	6.5 (1.8)	6.3 (2.1)	0.52	6.4 (2.0)	6.7 (2.0)	0.55
Perceived Motivation	8.3 (1.4)	8.8 (1.2)	7.9 (1.5)	**<0.01**	8.7 (1.2)	8.0 (1.5)	**0.01**	8.3 (1.5)	9.1 (1.0)	0.07
**Leadership statements, [n] (%)**										
All leadership statements	6.1 (6.2)	7.9 (7.8)	4.6 (3.8)	**<0.01**	6.1 (5.5)	6.1 (6.8)	0.95	5.4 (5.5)	11.9 (8.8)	**<0.01**
Secure statements	4.0 (4.7)	5.2 (5.8)	2.9 (3.1)	**<0.01**	4.3 (4.8)	3.6 (4.6)	0.45	3.4 (3.7)	9.0 (8.4)	**<0.01**
Insecure statements	2.0 (2.0)	2.3 (2.3)	1.6 (1.5)	0.07	1.6 (1.5)	2.3 (2.3)	0.08	1.9 (2.0)	2.5 (1.9)	0.38
**Critical decisions, [n] (%)**										
Person to execute FMM	23 (21.3%)	16 (32%)	7 (12%)	**0.01**	13 (26%)	10 (17%)	0.27	20 (21%)	3 (27%)	0.61
Person to start massage	23 (21.3%)	14 (28%)	9 (16%)	0.11	13 (26%)	10 (17%)	0.27	20 (21%)	3 (27%)	0.61
Person to start ventilation	23 (21.3%)	10 (20%)	13 (22%)	0.76	11 (22%)	12 (21%)	0.87	21 (22%)	2 (18%)	0.79
Person to start defibrillation	23 (21.3%)	10 (20%)	13 (22%)	0.76	12 (24%)	11 (19%)	0.52	18 (19%)	5 (45%)	**0.04**

FMM: first meaningful measure of resuscitation

Perceived stress did not show significant gender-differences in this study, neither did stress-overload index. Regarding personality traits, females had higher values of neuroticism (3.1 [±0.7] vs. 2.6 [±0.6], p<0.01). Male students made more leadership statements than their female colleagues (mean of 7.9 [±7.8] vs. 4.6 [±3.8], p<0.01). In particular, men made more secure statements compared to women (5.2 [±5.8] vs. 2.9 [±3.1], p<0.01). Finally, male students made more often critical decisions to initiate the resuscitation than female students (16 (32%) vs. 7 (12%), p = 0.01). All differences between male and female students also remained statistically significant after adjustment for leadership-designation.

To further assess the effect of self-esteem, a median split was performed in the Rosenberg self-esteem score allowing the comparison of individuals with low self-esteem versus high self-esteem. We found that individuals with low self-esteem had significantly lower motivation (mean of 8.0 [±1.5] vs. 8.7 [±1.2], p = 0.01) and made more insecure leadership statements than participants with high self-esteem without reaching statistical significance (2.3 [±2.3] vs. 1.6 [±1.5], p = 0.08). Regarding personality traits, individuals with high self-esteem showed more openness to experience (4.4 [±0.6] vs. 4.1 [±0.6], p = 0.02). In contrast, individuals with low self-esteem had more neurotic personality traits (3.2 [±0.7] vs. 2.5 [±0.6], p<0.01). Perceived stress and stress-overload did not differ significantly between individuals with high versus low self-esteem.

Finally, we compared the randomly designated team-leaders with the remaining participants. We found that the designated leaders differed from other participants in terms of number of leadership statements and number of critical decisions (defibrillation) but not in terms of self-esteem or personality traits (see Table 4).

## Discussion

This study investigating the effect of psychological variables on resuscitation performance and stress levels of medical students during a simulated cardiac arrest has several findings that are worth to be mentioned.

First, we found that mean levels of self-esteem within groups was the only psychological variable to predict team performance during resuscitation. In fact, self-esteem was significantly associated with hands-on time. Also, in a subgroup analysis, individuals with high self-esteem were significantly more motivated during the resuscitation scenario compared to individuals with low self-esteem.

Second, we found that mean team self-esteem was an important confounder for the association between other psychological variables and CPR performance. Specifically, mean team conscientiousness showed a significant association with team performance in a multivariate analysis adjusted for group size and leadership designation, but this result was no longer significant when also adjusting for mean team self-esteem. Albeit not reaching statistical significance, the association of mean team stress-overload as well as perceived stress with performance was also reduced in models including self-esteem. This again suggests that the effect of perceived stress on hands-on time is potentially mediated by self-esteem. While higher mean team stress-overload and perceived stress were shown to negatively impact CPR performance during simulated resuscitation in previous research, our results suggest that this effect might be confounded by self-esteem and that stress alone might have less impact on CPR performance as previously assumed [1, 45]. Further research has shown that self-esteem facilitates persistence after failure and leads to better job performance [13].

Previously reported associations between low self-esteem and cardiovascular stress parameters could not be replicated in our study, as we observed no significant effect of self-esteem on ECG stress measures [38, 40]. In addition, perceived stress showed no association with objective stress markers such as HR or HRV, indicating that physical stress through chest compression or general physical activity during resuscitation may be a confounder of these physiological markers. In fact, it is known that HR and HRV vary with both psychological and physical stress[32, 46]. Alternatively, the small number of subjects included limited the power of our analysis (Type II error) and in a larger sample these associations may become significant.

Fourth, past research has shown that leadership is crucial to CPR performance[14]. Consequently, we were interested in analysing psychological variables of group leaders and their effect on team performance. We found that teams in which leaders had an agreeable personality showed a significant better performance in regard to hands-on time. This finding is in line with earlier research, where agreeableness predicted leadership statements[15]. Also, studies from the field of occupational psychology demonstrate that agreeableness favours social interactions [15, 44, 47, 48]. Interestingly, groups of team leaders with high levels of extraversion did not show improved performance, as we initially expected. In fact, extroverted leaders made less secure leadership statements and CPR was initiated later when compared to other teams. These findings are in contradiction with previous research, which suggested that extraversion predicts more leadership statements [15].

Researchers generally agree on the point that extraversion and conscientiousness are strong predictors of leadership emergence [8]. Extraverted individuals are often perceived as leader-like, and extraverted leadership results in higher perceived team efficacy and performance [8, 49]. However, our results suggest that objective leadership effectiveness may be rather decreased by extroverted leaders. While the mean self-esteem level in the group was an important predictor of hands-on time, self-esteem in designated leaders was not associated with team performance. Thus, leader’s personality (e.g., agreeableness) seems to have a more persistent impact on team performance even after adjusting for self-esteem, while the opposite was found on a team level.

Finally, we analysed gender differences in psychological variables, performance and leadership. In 2017, Amacher et al. found that female students showed less leadership behaviour in a simulated resuscitation, which was associated with lower performance of female students compared to male students [22]. In the current study, we again found that female participants made less leadership utterances compared to males. In line with previous work, we also found that female students reported lower self-esteem compared to males [50]. Perceived stress however was similar between genders. As our study was conducted with mixed groups in regard to gender composition, we cannot make any strong conclusion about this issue. Still, the differences in self-esteem between males and females may explain some of the gender differences found in previous studies[15, 22].

We are aware of several limitations to this study. First, the setting of our study is a simulated cardiac arrest and not a real clinical scenario. However, previous studies showed that simulated cardiac arrests in high fidelity simulators are experienced as realistic and stressful, similar to real clinical scenarios [51, 52]. Second, all of our participants were medical students from the same year of the University of Zurich undergoing the identical curriculum limiting the generalisability of our results to more experienced healthcare providers such as staff working in emergency or intensive care settings. Third, our study was performed in either teams of three or six students, possibly biasing individual behaviour during the simulation, as individuals in smaller groups are more likely to be challenged and involved in the resuscitation scenario. Fourth, we used the Big-5 personality questionnaire to assess the personality traits of group leaders. As leaders were not rated by others but evaluated themselves, results of this test might be biased. Fifth, the video-recordings were only coded by one person. However, we held repetitive meetings to discuss ambiguities until consensus was found. Sixth, we assessed self-esteem of students as a possible predictor of resuscitation performance. Self-esteem may depend on several cultural and personal factors (e.g. gender, age) and may not be easy to influence. Still, future studies should investigate the effect of specific interventions (e.g. CPR trainings) on self-esteem and CPR performance [50]. Seventh, we used the combined variable “stress-overload” to investigate associations of perceived stress and different outcome measures, which was based on prior work from our group [4]. The absence of significant association may also relate to the fact that there is no optimal measure of perceived stress in a simulated medical emergency situation where traditional stress markers may be influenced by physical activity of students. Finally, the small sample size may have caused type II error and validation in a larger sample would be important. We expect some of our results to become statistically significant when tested in a larger sample.

## Conclusion

In this simulator study, we found that psychological variables including self-esteem and agreeableness of team leaders of inexperienced students were associated with cardiopulmonary resuscitation performance. Whether enhancing these factors during resuscitation trainings serve for better performance remains to be studied.

## Supporting information

S1 Data (XLS)
